# Perineuronal Nets in Syrian Hamsters: Anatomical Localization, Sex Differences, Diurnal Variation, and Response to Social Stress

**DOI:** 10.1002/brb3.70189

**Published:** 2024-12-22

**Authors:** Emma K. Shaughnessy, Benjamin W. Horne, Kim L. Huhman

**Affiliations:** ^1^ Neuroscience Institute Georgia State University Atlanta Georgia USA

**Keywords:** agonistic behavior, circadian, PNN, social defeat, WFA

## Abstract

**Purpose:**

Perineuronal nets (PNNs) are extracellular matrix proteoglycans surrounding neurons and glia. It has been suggested that PNNs are involved in the pathophysiology of multiple CNS illnesses, including stress‐related neuropsychiatric disorders like schizophrenia, major depressive disorder, and anxiety disorders.

**Method:**

Before examining the putative role of PNNs in stress‐related responses, we described for the first time the anatomical distribution in Syrian hamsters (*Mesocricetus auratus*), an excellent model organism for studying social stress and circadian rhythms.

**Results:**

We observed PNNs throughout the hamster cortex and hippocampus but found low to no expression in subcortical regions such as the hypothalamus, thalamus, and striatum, sites where they are observed in rats and mice. We further demonstrated that PNNs are dynamically regulated in a sex‐dependent manner in response to acute social stress, specifically in hippocampal area CA1. We did not observe a difference in PNNs between the beginning of the dark versus light phase of the light–dark cycle in hamsters, despite other laboratory rodents showing diurnal variation in PNNs. Finally, we also demonstrated that there are sex differences in PNN expression in the somatosensory cortex and the basolateral amygdala in hamsters, suggesting that sex as a biological variable should be considered in studies of PNN function.

**Conclusion:**

Together, the data from the current study suggest that a comparative approach will be necessary to fully elucidate the functional role of PNNs and, further, that Syrian hamsters are a valuable model in this endeavor.

## Graphical abstract

Syrian hamsters exhibit an anatomical distribution of perineuronal nets (PNNs) that is unique from that observed in other common laboratory rodents. We observed little change in PNNs based on lighting or after social defeat stress, but we did find significant sex differences in PNNs, indicating that studies of PNN function should include both sexes (figure shows 10× magnification of *Wisteria floribunda* agglutinin [WFA] immunoreactivity in hamster brain, including somatosensory cortex [SSC], medial septum [MS], and motor cortex [MC].

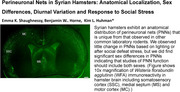

## 1 Introduction

Perineuronal nets (PNNs), which are extracellular matrix proteoglycans that surround neurons and glia, were traditionally thought to act only as structural support in the nervous system. Although their anatomical distribution throughout the central nervous system is not well characterized across species, the existing data suggest that PNNs primarily surround inhibitory neurons (Seeger et al. 1994). In rhesus macaques, PNNs are mainly seen surrounding cells in the cortex and are largely absent from subcortical regions such as the hypothalamus and caudate/putamen (Mueller et al. 2016). However, in mice, PNN expression occurs throughout subcortical structures, including multiple thalamic nuclei, the lateral and medial globus pallidus, and the anterior hypothalamus (Ciccarelli et al. 2021). Human studies have largely focused on expression in the cortex, hippocampus, or amygdala (Pantazopoulos et al. 2010; Rogers et al. 2018), with little additional information available regarding PNN distribution in other brain regions.

As it began to be recognized that these molecules were more labile than previously believed, PNNs were studied during postnatal development and were shown to play a role in controlling neuronal plasticity (Sorg et al. 2016) and in regulating the opening and closing of so‐called critical periods of development. For example, the ontogeny of PNNs is correlated with the crystallization of song learning in zebra finches (Cornez et al. 2018). More recently, Banerjee et al. (2017) showed that PNNs are dynamically regulated in adults following fear conditioning, suggesting that they may play an important role in learning and memory. In addition, PNNs appear to vary across the light–dark cycle in mice and rats (Pantazopoulos et al. 2020) and may be sexually dimorphic in mice in brain regions known to modulate reproductive behavior (Harkness et al. 2021). Recent studies have also implicated PNNs in the etiology of multiple neuropsychiatric illnesses, including mood and anxiety disorders (Sorg et al. 2016; Browne et al. 2022), and the current conception of PNNs as being more plastic than originally thought is consistent with recent evidence that PNNs change in response to stress and inflammation (Bosiacki et al. 2019) (for review see Sorg et al. 2016). Thus, PNNs are emerging as a new mechanism in the modulation of complex behavior and possibly in the development of stress‐related neuropsychiatric disease, underscoring the importance of examining PNNs in a range of models so that we can better understand their distribution and potential role in modulating behavior. The initial purpose of the present study was to characterize for the first time the distribution of PNNs in Syrian hamsters, which display a rich array of social and agonistic behaviors (Albers, Huhman, and Meisel 2002; Albers et al. 2006).

Exposure to stress in humans is known to be an important risk factor for developing mood and anxiety disorders and to induce or exacerbate symptoms of these mental illnesses (Kessler et al. 2003). Millions of Americans are affected by these debilitating diseases, and roughly one‐third of patients are resistant or respond suboptimally to standard antidepressant medications (Kessler et al. 2003; Huh et al. 2011). Thus, there is a critical need to identify novel targets for the development of alternative treatments. Social stress, and social defeat in particular, is arguably the most common and salient type of stress experienced by humans and nonhuman animals (Almeida 2013; Bjorkqvist 2001; Huhman 2006). Both humans and nonhumans exposed to social defeat stress subsequently display increased anxiety‐ and depression‐like signs and symptoms, including social avoidance (Bjorkqvist 2001; Huhman 2006; Wood and Bhatnagar 2015; Toyoda 2017). Much like fear conditioning, behavioral responses to social defeat stress require learning and adaptation to environmental challenges, processes that thus might involve the PNNs (Huhman 2006; Jasnow et al. 2005; Day et al. 2011).

Syrian hamsters readily exhibit aggression and territorial behaviors in the laboratory and rapidly establish dominant‐subordinate relationships between dyads. Because their aggression is highly ritualized, it rarely results in physical injuries, making it possible to study the effects of social defeat stress without the confound of injury and the accompanying inflammation. In addition, both sexes display similar agonistic behavior, so it is possible to evaluate sex differences in the response to social defeat stress. Therefore, we tested the hypothesis that PNNs vary following social defeat stress and, furthermore, that they might do so in a sex‐dependent manner (Huhman 2006; Huhman et al. 2003; Solomon et al. 2006). Finally, we have recently observed a marked variation in the response to a pharmacological treatment aimed at reducing the behavioral response to defeat when the treatment was given at the beginning of the dark (active) phase versus at the beginning of the light (inactive) phase of the daily cycle (unpublished data). Because other rodent species show diurnal variation in PNN expression (Pantazopoulos et al. 2020; Harkness et al. 2021), it is thus possible that variation in PNNs could underlie daily variation in behavior or in response to pharmacological treatments. Given that hamsters exhibit more dramatic daily variation in behavior than do many laboratory rat and mouse species (Albers et al. 1984; Burgoon, Lindberg, and Gillette 2004), hamsters might then be an ideal species within which to interrogate the possible mechanisms underlying daily variation in behavior and drug responses. Therefore, we also tested the hypothesis that there is a daily variation in PNNs in hamsters that might underlie, at least in part, the observed diurnal variation in drug response.

## 2 Methods

### 2.1 Animals

Male and female hamsters were bred in‐house using animals obtained from Charles Rivers Laboratories (New York, NY or Wilmington, MA). Hamsters were group‐housed in same‐sex groups of three to five at weaning in static polycarbonate cages (23 × 42 × 20 cm) with corn cob bedding, paper nesting material, plastic tubes for environmental enrichment, and wire tops in a colony room on a 14:10 light/dark cycle, as is common in hamsters, to maintain gonadal patency (Ottenweller et al. 1987). Food and water were available ad libitum. All behavioral testing occurred between Postnatal Day (PND) 60 and 75, whereupon animals weighed between 110 and 160 g. Resident aggressors (RAs; > 170 g) were singly housed, an older male and ovariectomized female hamsters that were known to reliably attack a same‐sex intruder introduced in their home cage. Subjects were singly housed (23 × 42 × 20 cm) a minimum of 8 days before behavioral testing and gently handled each day leading up to behavioral testing to habituate them to experimenters. Syrian hamsters are thought to be territorial in the wild, and singly housing them does not have deleterious effects on their behavior or overall health (Ross et al. 2017). Estrous cycles for females were monitored by vaginal swabs for at least 8 days before testing, as described in detail by Chanut and Williams (2015). All females experienced their final or only defeat on Diestrus Day 1. All procedures and protocols were approved by the Georgia State University Institutional Animal Care and Use Committee and were in accordance with the standards outlined in the National Institutes of Health Guide for Care and Use of Laboratory Animals.

### 2.2 Experimental Design

#### 2.2.1 Experiment 1

Six male hamsters were transcardially perfused with 4% paraformaldehyde, as described below, within the first 2 h of the onset of the light phase of the daily cycle. Brains were harvested, and sectioned coronally on a CM3050 S cryostat (Leica Biosystems, Deer Park, MI) at 40 µm into four series. Histochemistry was performed on one of the series through the forebrain from +3.8 mm anterior to bregma −2.6 mm posterior to bregma to visualize the binding of fluorescein‐labeled *Wisteria floribunda* agglutinin (WFA) (Vector Labs, Burlingame, CA), as detailed below. Whole brain sections were imaged using *x*–*y* stitching on a Keyence microscope (Osaka, Japan) and evaluated for the number of labeled cells and density of staining in the forebrain, described in detail under Image Analysis below. All images for quantification were taken on the same day with identical imaging settings.

#### 2.2.2 Experiment 2

**FIGURE 1:**
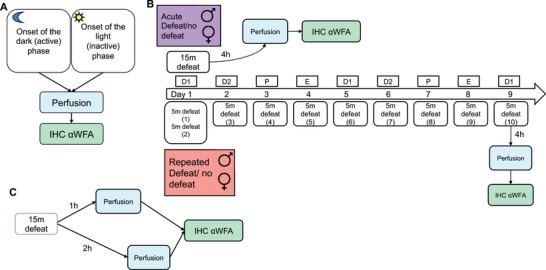
Experimental design for Experiments 2–4. Note that in Experiment 1, there were no experimental manipulations other than the collection of brains, so the design is not shown here. (A) Experiment 2 tested whether there are differences in PNN expression at the beginning of the dark (active) phase versus the beginning of the light (inactive) phase of the daily light–dark cycle. (B) Experiment 3 tested whether PNN expression is altered 4 h after acute or repeated defeat versus no defeat. All females were defeated for the first time on Diestrus Day 1 (D1) and were sacrificed on D1, as well. Animals receiving repeated defeat were defeated a total of 10 times over 9 days (the number of the defeat session is shown in parentheses). A similar number of males were tested each day with the females. (C) Experiment 4 was a small pilot to determine whether PNN expression might be altered 1 or 2 h after an acute defeat stressor. D2, Diestrus Day 2; E, estrus; P, proestrus.

The experimental design for Experiments 2–4 is shown graphically in Figure 1.

In Experiment 2, 12 males were transcardially perfused at either the onset of the light phase of the light–dark cycle or the onset of the dark phase (*n* = 6/time period), as described below. These times were chosen because we observed an opposite effect at these times of a drug treatment aimed at reducing the behavioral response to social defeat (unpublished data). Brains were harvested, sectioned coronally, and a histochemistry with fluorescein‐labeled WFA was performed. Slides were imaged on a Keyence microscope, and cells were counted using the FIJI Cell Counter tool (Schindelin et al. 2012), described in detail below. Brain regions were selected a priori based on results from Experiment 1 (regions showing high expression of WFA+ cells: the CA1 region of the hippocampus and the somatosensory cortex [SSC]) as well as regions that have been identified as being required for the behavioral response for social defeat (prefrontal cortex and its subregions, anterior cingulate cortex, prelimbic cortex, and infralimbic cortex, as well as the nucleus accumbens [NAc] and basolateral amygdala [BLA]).

#### 2.2.3 Experiment 3

A total of 20 males and 20 females were defeated either acutely or repeatedly (described below) and were sacrificed 4 h after their last defeat (or no defeat) session via transcardial perfusion. The 4 h delay was chosen because this was when there was a maximal difference in PNN expression observed in rats following fear conditioning (Banerjee et al. 2017). Brains were harvested, sectioned coronally, and a histochemistry was performed with WFA. The number of WFA+ cells was counted using the FIJI Cell Counter tool in the same brain regions as above.

#### 2.2.4 Experiment 4

A total of 18 males were subjected to a single acute social defeat session or to a no‐defeat control session, as described below. Either 1 or 2 h after defeat, animals were transcardially perfused with paraformaldehyde. Brains were harvested and sectioned. Histochemistry with WFA was performed, and the number of WFA+ cells was counted in the brain regions described above using the FIJI Cell Counter tool.

### 2.3 Social Defeat Training

Acute social defeat was performed as described previously in Huhman et al. (2003). Briefly, subjects were placed in the home cage of a same‐sex RA for 15 min and allowed to interact freely with the RA, which generally attacks an intruder within 30 s of the beginning of the trial. No‐defeat controls were placed in an empty RA cage for 15 min to control for handling and novel cage exposure. For repeated defeat, subjects were placed in the home cage of a novel RA for 10 sessions (5 min/trial) across 9 days to ensure that the females started and ended on Diestrus Day 1. On the first day, subjects experienced two defeat sessions with an intertrial interval of at least 45 min. For the light/dark experiments, the same protocol was used for acute defeat, but the defeats took place either within 2 h of the onset of the dark (or active) phase of the daily cycle or within 2 h of the onset of the light phase.

### 2.4 Visualization of WFA Staining

Animals were anesthetized with an overdose of sodium pentobarbital. Once deeply anesthetized, animals were transcardially perfused with cold phosphate‐buffered saline (PBS) and then 4% paraformaldehyde in phosphate buffer (pH 7.4). Brains were collected and stored in paraformaldehyde for 24 h, then moved to 30% sucrose for cryoprotection for a minimum of 3 days at 4°C. Brains were sectioned on a Leica CM3050 S cryostat (Deer Park, IL) into 40 µm sections and stored in cryoprotectant (30% sucrose and 30% ethylene glycol in PBS) at −20°C. Sections were washed in PBS five times and then blocked in 5% normal donkey serum in PBS with 0.1% Triton‐X for 1 h. They were then incubated with WFA with a conjugated fluorescein tag (Vector Labs, Burlingame, CA) overnight at 4°C. Sections were then washed three times in PBS, mounted to slides, and coverslipped with VectaShield Hardset with DAPI (Vector Labs, Burlingame, CA). WFA binds with specificity to the *N*‐acetylglucosamine epitope and is frequently used to stain PNNs throughout the brain. PNNs are heterogeneous, but most appear to express this epitope within the brain (Brückner et al. 1993).

### 2.5 Image Analysis

All images were taken with a Keyence microscope (Osaka, Japan) at 4×. Original image files were opened with FIJI, and the Cell Counter tool was used to count cells in each region, as determined by the Golden Hamster Brain Atlas (Morin and Wood 2001). In Experiment 1, bilateral cell counts were done in each brain region of interest (ROI) in each section, and an average of the counts for each brain region was calculated for each animal. Finally, an overall average was determined for WFA+ staining in each brain region. In Experiments 2–4, three sections from each animal were taken, and staining in each ROI was counted bilaterally. The average of all six counts was reported for each ROI. The medial prefrontal cortex (mPFC) (ACC, PL, and IL) sections were taken between 2.9 and 2.6 mm anterior to bregma. The SSC and hippocampal area CA1 sections were taken between −1.2 and −1.5 mm posterior to bregma. The BLA sections were from −0.9 and −1.2 mm posterior to bregma. Due to the size and variability of the SSC, a 1 × 1 mm^2^ was superimposed over the image, and only cells within that block were counted. The experimenter was blinded to the experimental condition during analysis. For anatomical distribution of PNNs, entire sections were imaged at 4× and stitched using Keyence BZ‐X software (Osaka, Japan). The criteria for scoring were as follows: − (negative or no) indicates 0–5 WFA+ cells and no staining in the neuropil, + (low) shows some somatic staining (> 5 but < 10 cells/mm^2^) and diffuse staining of the neuropil, ++ (moderate) indicates more intense somatic staining with a cell count between 10 and 20 cells/mm^2^, +++ (high) shows higher somatic staining with cell counts of 20 or more cells/mm^2^.

### 2.6 Statistical Analysis

Data were first analyzed for normality using Shapiro–Wilk's test and homogeneity of variance using Levene's test. One‐way ANOVAs with Fisher's LSD post hoc tests and Student's *t*‐test were performed where appropriate. Effect sizes were calculated using Cohen's *d* in Excel. A large effect size was ascribed at *d* ≥ 0.8, a medium effect size was *d* between 0.2 and 0.8, and a small effect size was *d* ≤ 0.2. Statistical analysis was performed using GraphPad Prism (9.1.2). All graphs were created in GraphPad Prism (9.1.2).

## 3 Results

### 3.1 Experiment 1: Anatomical Localization of PNNs in Male Hamsters

**FIGURE 2:**
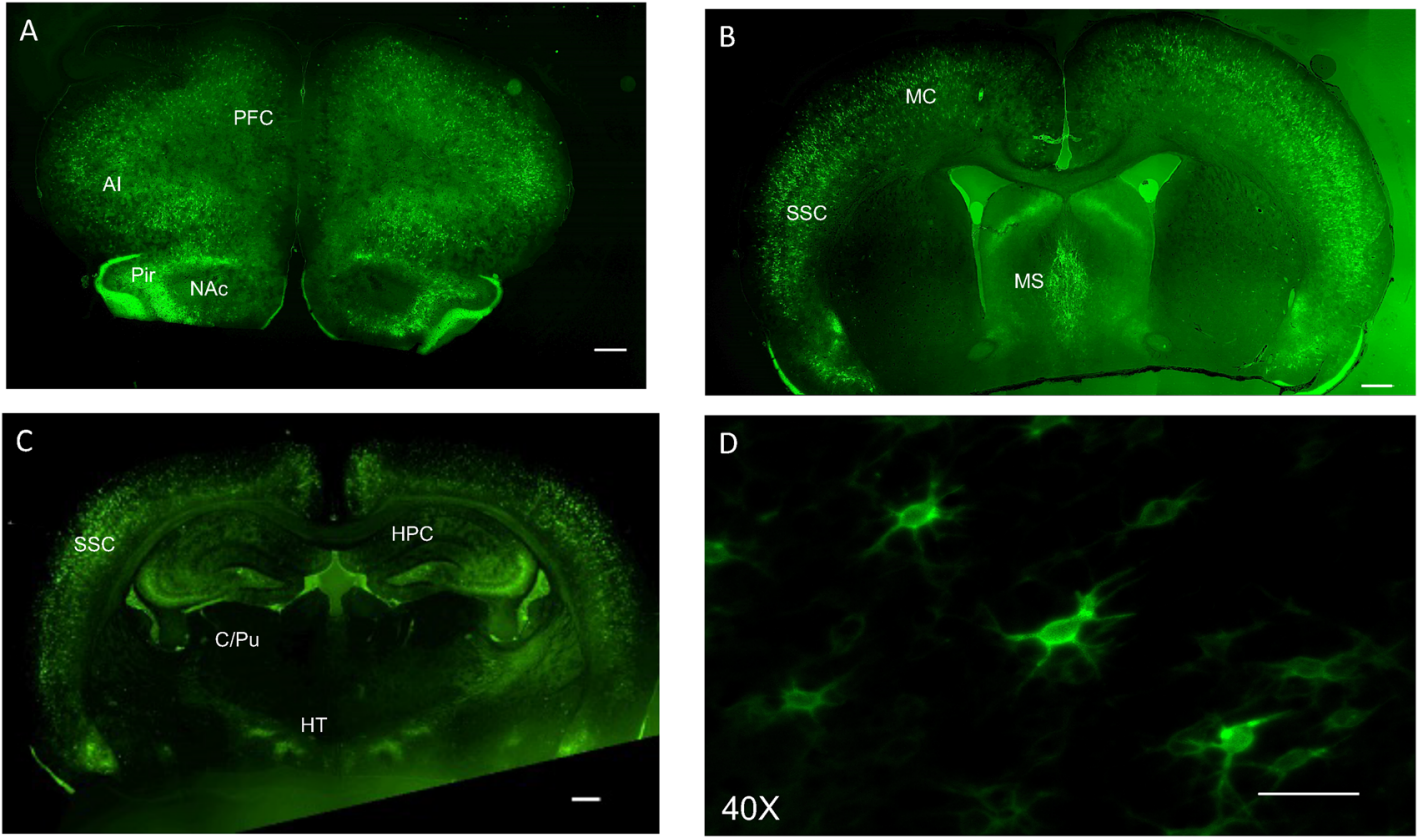
Representative micrographs showing WFA staining (green) in Syrian hamster brain (*n* = 6). (A–C) are taken at 4×, and the scale bar represents 500 µm. (A) The agranular insular cortex (AI) and piriform cortex (Pir) show high expression of WFA+ cells with moderate expression in the prefrontal cortex (PFC) and little to no apparent expression in the nucleus accumbens (NAc) (+3.22 mm AP from bregma as shown in the stereotaxic atlas of the hamster brain (Morin and Wood 2001). (B) Both the motor cortex (MC) and somatosensory cortex (SSC), as well as the medial septum (MS), show high expression of WFA+ cells (+1.8 mm from bregma). (C) The somatosensory cortex (SSC) and hippocampus (HPC) show high expression of WFA+ cells, while the hypothalamus (HT) and caudate/putamen (C/Pu) show little to no apparent WFA+ cells (−0.9 mm from bregma). (D) Higher magnification (40×) of perineuronal nets surrounding soma and proximal dendrites of cells in the mPFC (scale bar = 50 µm).

To date, no one has characterized where PNNs are expressed in hamster brains. We observed the highest expression of WFA binding in cortical regions, particularly the somatosensory and motor cortices (high expression) (Figure 2a; Table 1). The medial septum also showed high WFA+ staining, while the lateral septum showed low staining (Figure 2b). The basal ganglia, thalamus, and most of the hypothalamus showed little to no expression of PNNs. The hippocampus showed moderate expression in all subregions. The subregions of the amygdala showed no apparent expression of WFA (Figure 2c). The staining pattern of WFA in Syrian hamsters showed the same mesh‐like pattern surrounding soma and proximal dendrites that has been observed in other rodents and in humans (Figure 2d).

**TABLE 1:** Perineuronal net expression in Syrian hamster brain nuclei as labeled by WFA. − Indicates 0–5 WFA+ cells and no staining in the neuropil, + indicates < 10 WFA+ cells/mm^2^ and diffuse staining in the neuropil, ++ indicates more intense somatic staining with a cell count between 10 and 19 WFA+ cells/mm^2^, and +++ indicates higher somatic staining with cell counts of 20 or more WFA+ cells/mm^2^.

Brain region	Relative expression
Cortex	
Primary somatosensory	+++
Agranular insular cortex	++
Granular insular cortex	++
Motor cortex	+++
Retrosplenial granular cortex	++
Retrosplenial agranular cortex	+
Endopiriform nucleus	+++
Hippocampus	
Dentate gyrus	++
CA1	++
CA2	+
CA3	+
Hypothalamus	
Medial preoptic nucleus	+
Zona pncerta	++
Paraventricular nucleus	−
Anterior hypothalamus	−
Posterior hypothalamus	−
Arcuate	−
Thalamus	
Mediodorsal	−
Central medial	−
Centrolateral	−
Septum	
Medial septum	+++
Lateral septum	+
Striatum	
Caudate	−
Putamen	−
Globus pallidus	−
Nucleus accumbens	−
Amygdala	
Medial	−
Central	+
Basolateral	−

### 3.2 Experiment 2: Diurnal Variation in PNNs Was Not Observed in Male Hamsters Within Selected Brain Regions That Are Known to Mediate Behavioral Responses to Social Stress

In C57BL/6 mice, PNNs appear to be dynamically regulated across the light–dark cycle in mPFC, habenula, amygdala, and all subregions of the hippocampus (Pantazopoulos et al. 2020). Sprague‐Dawley rats also show variation in PNN expression across the light–dark cycle in the mPFC (Harkness et al. 2021). As noted above, we have observed different behavioral outcomes when drugs were given at the onset of the light or dark phases of the daily cycle. A result that we hypothesized could be based, at least in part, on variation in PNNs at these time points. Therefore, we explored whether PNNs in Syrian hamsters differ between the onset of the light and the onset of the dark phases. Males were sacrificed at the onset of the light phase or the dark phase of the light–dark daily cycle, and the number of PNNs in selected brain regions was counted. The brain regions selected were those that are part of the putative Social Behavior Neural Network (Newman 1999; Albers 2012) and that we have previously determined are necessary for the acquisition or expression of behavioral responses to social defeat (Markham, Luckett, and Huhman 2012; Luckett, Norvelle, and Huhman 2012; Markham, Taylor, and Huhman 2010)—the BLA, NAc, and subregions mPFC (anterior cingulate cortex, prelimbic cortex, and infralimbic cortex; ACC, PL, and IL, respectively) as well as areas showing high expression of PNNs in naïve animals—SSC and CA1 region of the hippocampus (CA1). BLA was selected before the study was conducted; when Experiment 1 showed no staining in the BLA (< 5 cells), we still included BLA in Experiments 2 and 3 because previous studies have shown behavioral changes can occur even when only a few BLA cells have been manipulated (Jasnow et al. 2005).

**FIGURE 3:**
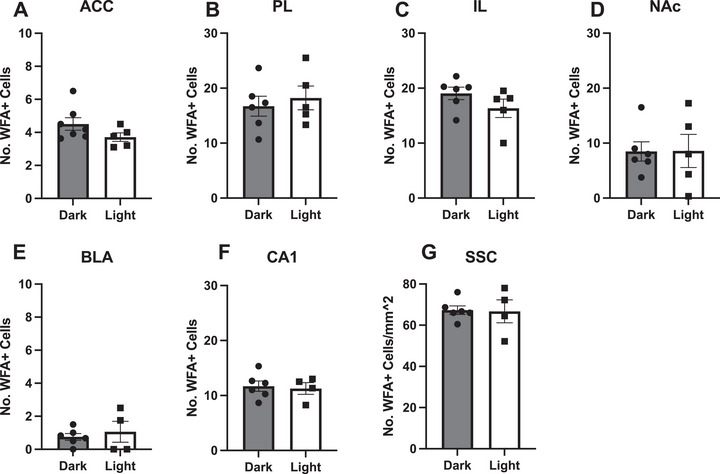
The number of WFA+ cells does not appear to differ between the beginning of the dark (active) versus the light phase of the daily light–dark cycle in any of the brain regions studied (*n* = 12 hamsters). Similar expression of WFA+ cells was observed in the dark (gray bars with circle dots) and light (white bars with square dots) in the anterior cingulate cortex (A), prelimbic (B), and infralimbic cortices (C), nucleus accumbens (D), basolateral amygdala (E), hippocampus (F), and somatosensory cortex (G). Note the scale differences between regions. See Table 1 for quantification of PNNs in specific brain regions.

None of the areas examined showed differential expression based on lighting conditions (Figure 3) as determined by Student's *t* tests: ACC *t* = 1.575, *p* = 0.1464, *d* = 0.9693 (Figure 3a); PL *t* = 0.5347, *p* = 0.6058, *d* = 0.3222 (Figure 3b); IL *t* = −1.3755, *p* = 0.2022, *d* = 0.8193 (Figure 3c); NAc *t* = 0.02905, *p* = 0.9775, *d* = 0.0172 (Figure 3d); BLA *t* = 0.5562, *p* = 0.5933, *d* = 0.3248 (Figure 3e); CA1 *t* = −0.2941, *p* = 0.7762 (Figure 3f); SSC *t* = −0.1127, *p* = 0.9130 (Figure 3g). Despite not reaching significant *p* values, large effect sizes were observed in ACC and IL, and medium effect sizes were observed in PL and BLA.

### 3.3 Experiment 3: Hippocampal Area CA1 Shows Significant Changes in WFA+ Staining After Defeat Stress; WFA+ Staining Shows Marked Sex Differences in Naïve and Stressed Animals

**FIGURE 4:**
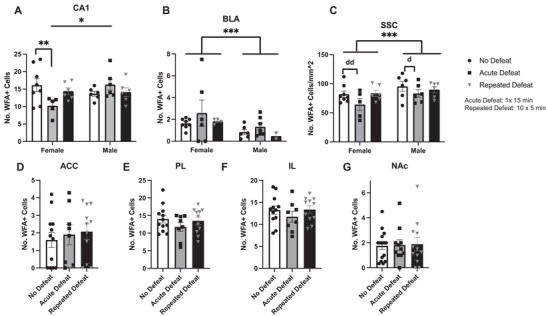
Number of WFA+ cells in select brain regions after no defeat (white bars with circles), acute defeat (light gray bars with squares), or repeated defeat (dark gray bars with triangles). (A) Hippocampal area CA1 (CA1) shows an interaction between sex and defeat status (*F*(2,31) = 4.629, *p* = 0.0174), with defeat appearing to reduce the number of WFA+ cells in females but increase them in males, particularly after acute defeat. (B) In the basolateral amygdala (BLA), females display higher expression of PNN cells compared to males regardless of defeat status (significant main effect of sex *F*(1,27) = 4.596, *p* = 0.0412). (C) In the somatosensory cortex (SSC), conversely, males have higher expression of PNNs compared to females regardless of defeat status (significant main effect of sex *F*(1,31) = 5.319, *p* = 0.0279). (D–G) There was no significant effect of sex on WFA+ cells in the anterior cingulate cortex (ACC), prelimbic cortex (PL), infralimbic cortex (IL), and nucleus accumbens (NAc); thus, the data were collapsed over sex in these brain regions. There was also no significant effect of defeat in these brain regions (*n* = 20 male and 20 female hamsters), * denoted significant interaction of defeat status by sex; ** denotes acute defeat is significantly less than no defeat (Fisher's LSD, *p* < 0.05); *** denotes the significant effect of sex regardless of defeat status; “d” indicates a medium effect size and “dd” indicates a large effect size (Cohen's *d*; as defined in Section 2.6).

Because PNNs are dynamically regulated 4 h after fear conditioning (Banerjee et al. 2017), it was hypothesized that PNNs would be similarly altered following social defeat stress. In CA1, we observed a significant interaction of sex and defeat status (Figure 4a: Interaction *F*(2,31) = 4.629, *p* = 0.0174; Sex *F*(1,31) = 0.8960, *p* = 0.3512; Defeat timing *F*(2,31) = 0.6469, *p* = 0.5306), with acute defeat slightly increasing PNNs in males (not statistically significant) but decreasing them in females (Fisher's LSD *p* = 0.0064). In the BLA (Interaction *F*(2,27) = 0.1284, *p* = 0.88; Sex *F*(1,27) = 4.596, *p* = 0.0412; Defeat timing *F*(2,27) = 1.147, *p* = 0.3326) and the SSC (Interaction *F*(2,31) = 0.4015, *p* = 0.6727; Sex *F*(1,31) = 5.319, *p* = 0.0279; Defeat timing *F*(2,31) = 2.702, *p* = 0.0829), there was no change in the number of WFA+ cells after acute or repeated defeat, but we did observe a significant sex difference (Figure 4b,c) in these regions. As shown in Figure 4d–g, there was no significant effect of defeat or sex on the number of WFA+ cells, so data were collapsed across sex on the graphs (ACC [Interaction *F*(2,25) = 0.4271, *p* = 0.6571; Sex *F*(1,25) = 1.813, *p* = 0.1902; Defeat timing *F*(2,25) = 0.2529, *p* = 0.7785]; PL [Interaction *F*(2,25) = 0.2652, *p* = 0.7692; Sex *F*(1,25) = 0.8557, *p* = 0.3638; Defeat timing *F*(2,25) = 0.8882, *p* = 0.4240]; IL [Interaction *F*(2,25) = 0.1654, *p* = 0.8485; Sex *F*(1,25) = 0.001399, *p* = 0.9705; Defeat timing *F*(2,25) = 0.6757, *p* = 0.5178]; NAc [Interaction *F*(2,32) = 0.5564, *p* = 0.5787; Sex *F*(1,32) = 0.01099, *p* = 0.9172; Defeat timing *F*(2,32) = 0.02839, *p* = 0.9720]). Of note, although there were no significant differences among groups in the SSC, there was a large effect size of acute defeat versus no defeat in females (Figure 4c; *d* = 0.9125) and a medium effect size of acute defeat in males (*d* = 0.6626).

### 3.4 Experiment 4: PNN Expression Does Not Vary 1–2 h After an Acute Social Defeat Stressor

**FIGURE 5:**
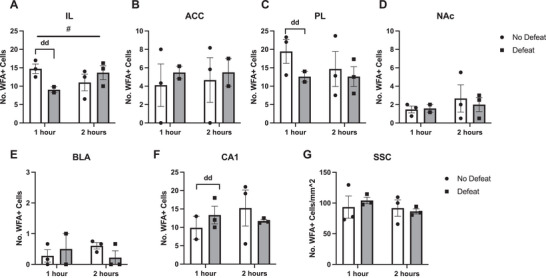
Number of WFA+ cells one and 2 h after a single social defeat (gray bars with squares) compared to no‐defeat controls (white bars with circles) (A) infralimbic cortex (IL) shows a trend that 1 h after an acute defeat, the number of WFA+ cells is reduced (*F*(1,7) = 5.067, *p* = 0.0591, Cohen's *d* = 3.1983) but rebounds 2 h after defeat. (B–G) The anterior cingulate cortex (ACC), prelimbic cortex (PL), nucleus accumbens (NAc), basolateral amygdala (BLA), CA1 region of the hippocampus, and the somatosensory cortex (SSC) do not show significant differences in WFA+ cells 1 or 2 h after defeat compared to no‐defeat controls (*n* = 18 hamsters). ^#^ *p* = 0.0591; “dd” denotes large effect size (Cohen's *d*; as defined in statistical analysis).

Because there were no changes in PNN expression 4 h after social defeat in a majority of the brain regions evaluated in Experiment 3, we examined the possibility that the 4‐h time point was too late to capture transient changes in PNN expression. A small pilot study was run, and animals were subjected to a single social defeat within the first 2 h of the onset of the dark phase of the light–dark cycle and sacrificed either 1 or 2 h after the defeat stressor. The number of PNNs in preselected brain regions was counted. None of the regions evaluated showed significant changes in the number of WFA+ cells 1 or 2 h after defeat compared to no‐defeat controls (Figure 5). However, there was a trend toward an interaction between defeat and time of sampling in CA1 (Interaction *F*(1,7) = 5.507, *p* = 0.0591; Time *F*(1,7) = 0.05927, *p* = 0.8146; Defeat status *F*(1,7) = 0.6607, *p* = 0.4431). A two‐way ANOVA was run for all brain regions examined. ACC: Interaction *F*(1,6) = 0.01434, *p* = 0.9086, Time *F*(1,6) = 0.01617, *p* = 0.9030, Defeat status *F*(1,6) = 0.2402, *p* = 0.6414; PL: Interaction *F*(1,7) = 0.4370, *p* = 0.5297, Time *F*(1,7) = 0.4270, *p* = 0.5343, Defeat status *F*(1,7) = 1.505, *p* = 0.2596; NAc: Interaction *F*(1,7) = 0.1582, *p* = 0.7027, Time *F*(1,7) = 0.6783, *p* = 0.4373, Defeat status *F*(1,7) = 0.08067, *p* = 0.7846; BLA: Interaction *F*(1,7) = 1.550, *p* = 0.2532; Time *F*(1,7) = 0.010057, *p* = 0.9210, Defeat status *F*(1,7) = 0.1098, *p* = 0.7501; CA1: Interaction *F*(1,7) = 1.136, *p* = 0.3218, Time *F*(1,7) = 0.3315, *p* = 0.5828, Defeat status *F*(1,7) = 4.074e−5, *p* = 0.9951; SSC: Interaction *F*(1,8) = 0.4776, *p* = 0.5090, Time *F*(1,8) = 0.6895, *p* = 0.4304, Defeat status *F*(1,8) = 0.05453, *p* = 0.8212. IL showed a large effect size between 1 h no defeat and 1 h defeat (Cohen's *d* = 3.1983), as did PL (Cohen's *d* = 1.626) and CA1 (Cohen's *d* = 0.8055).

## 4 Discussion

The present study is the first to characterize the distribution of PNNs in Syrian hamsters, a species that has been widely used in studies of agonistic and communicative behavior as well as of circadian rhythms (Albers, Huhman, and Meisel 2002, Albers et al. 2006; Albers 2012). We found that the anatomical distribution of PNNs in Syrian hamsters is somewhat different from that reported in other lab rodents, such as mice (*Mus musculus*) and rats (*Rattus norvegicus*). In hamsters, PNN staining is observed mainly throughout the cortex while being low or absent in the hypothalamus, thalamus, and striatum. In mice and rats, expression of PNNs is also highest in cortical regions but is also more widely spread throughout the brain, including subcortical structures like thalamic nuclei, amygdala, and striatum, compared to hamsters (Ciccarelli et al. 2021). Human studies on PNNs thus far have been restricted to preselected brain regions or ROIs within the cortex, hippocampus, or amygdala, so how the distribution of PNNs in hamsters compares to that in humans across the brain is yet to be determined. In marmosets, a nonhuman primate, the distribution of PNNs is also distinctly different than that observed in mice in many brain regions (Ciccarelli et al. 2021) but appears to more closely mimic the pattern observed in hamsters. Together, the data suggest marked differences in the regional expression of PNNs among species and suggest that the selection of model species could have an enormous impact on the conclusions drawn. Given that hamsters are more human‐like in their physiology and susceptibility to a variety of diseases than are many more commonly used animal models (for a review, see Fan et al. 2014), it may be that hamsters are a valuable model for asking questions about PNN function as well. The data certainly demonstrate that a comparative approach is going to be necessary to determine the range of functions subserved by PNNs.

We hypothesized that PNN expression would be different at different phases of the light–dark cycle based on literature examining PNN expression across the day in mice (Pantazopoulos et al. 2020). Contrary to this hypothesis, however, we observed no significant difference in PNN expression between the beginning of the dark, or active, phase of the cycle compared to the beginning of the light, or inactive, phase in male hamsters. Again, we chose these two time points because they are the times when we have observed behavioral differences in response to pharmacological manipulations in male hamsters. It is certainly possible that there could be daily changes in PNN expression that occur outside these two time points. Indeed, in mice and rats, wherein differences in PNN expression across the daily light–dark have been reported, significant differences were observed in the middle of the light or dark phase and not at the time of lighting transitions (Pantazopoulos et al. 2020). Future studies should further characterize the possible daily or circadian variation in PNN expression in hamsters across a wider range of times. Future studies across species should also determine if diurnal variation, if present, is a result of the daily light–dark cycle or if it is driven by the circadian clock. A limitation, which must be acknowledged given the sex differences discussed below, is that we included only males in this experiment. Future studies should determine whether there is diurnal variation in PNN expression in female hamsters. The current data, however, do not support our hypothesis that differences in PNN expression underlie behavioral differences that we have previously observed between the beginning of the dark versus light phases of the daily light–dark cycle.

In hamsters, PNNs do not appear to be particularly labile after social defeat stress, given that we observed no significant changes in PNN expression following defeat stress in most of the brain regions sampled. The only brain area in which we observed a significant effect of defeat on the number of PNN‐enwrapped cells was hippocampal region CA1, wherein there was a defeat by sex interaction. We have previously demonstrated that the hippocampus is required for the acquisition of behavioral responses to social defeat stress in Syrian hamsters (Markham, Taylor, and Huhman 2010), and c‐Fos mRNA expression increases in this brain region after handling stress (Kollack‐Walker, Watson, and Akil 1997). In addition, the hippocampus is required for recognizing novel and familiar conspecifics in hamsters (Lai et al. 2005). Given this, it is possible that the observed decrease in the number of WFA+ cells following defeat in females in the CA1 region of the hippocampus may contribute to neuronal plasticity or activity following exposure to stress. In addition, males may respond more strongly to social defeat than females (Huhman et al. 2003; Bath and Johnston 2009; Faruzzi et al. 2005). Thus, it is possible that the decrease in WFA+ cells in CA1 might contribute to the relative resilience of females to social defeat. This possibility could be tested in future studies. Most of the other brain regions studied have also been implicated in response to social defeat stress and yet fail to show changes in PNN expression. This could be due to the heterogeneity of cell types within these regions, especially within the cortical regions. A future study could examine the co‐expression of WFA with other cellular markers to determine the phenotype of cells associated with PNNs and to discover if PNNs associated with a subset of these are more labile.

Relatedly, this study used only WFA to quantify PNN expression. This is the most commonly used method for detecting PNNs (Härtig et al. 2022). PNNs, themselves, are heterogeneous, containing multiple different chondroitin sulfate proteoglycans (CSPGs) such as aggrecan, neurocan, and brevican (Galtrey and Fawcett 2007); however, WFA staining cannot differentiate between these CSPGs and stains anything containing an internal or terminal *N*‐acetylgalactosamine. Future studies may be useful in comparing PNN component expression to WFA‐labeled cells in Syrian hamsters to note any differences, but the data above provide a solid baseline for understanding the expression of PNNs throughout hamster brains.

It is also important to note that we observed interesting sex differences in PNN expression independent of defeat stress in the BLA and SSC, with females exhibiting increased expression in the BLA and decreased expression in the SSC compared to males. This finding emphasizes the need to examine PNN staining in both sexes and suggests that PNNs may play a role in mediating neuronal sex differences in regions of the brain that are important for controlling social behavior.

Our final experiment was a small pilot to determine whether the selection of the 4 h time delay was perhaps too late to observe potential differences in PNN expression. That time window was justified by the existing literature (Banerjee et al. 2017), but the time course of PNN responses has certainly not been studied exhaustively. Thus, our pilot study examined two additional time points following defeat. Despite the comparisons being underpowered, we observed a trend in IL toward a decrease in PNN expression at 1 h that had rebounded by 2 h after defeat, and there were medium to large effect sizes of defeat in some of the other brain regions, such as the infralimbic and prelimbic cortices. For the most part, however, the “time course” experiment suggested that the absence of widespread changes in PNNs following defeat in Experiment 3 was not due to the timing of sampling. Of course, it is entirely possible that we still missed the ideal time point or that PNNs change significantly in brain regions that we did not examine. The current data do suggest that further studies investigating the time course of PNN expression after social defeat may be warranted. Interestingly, it should also be noted that PNNs have been shown to exhibit hemispheric differences in response to behaviors such as maternal care (Lau et al. 2020), suggesting the possibility that we could have missed effects of defeat or sex on PNNs that are hemisphere‐dependent. Future studies could thus evaluate hemispheric differences after social defeat stress.

Although most of the experiments in the current study included only males, it is important to note that we did include both sexes in Experiment 3, which revealed that there may be important sex differences in the number of PNN‐enwrapped cells in the BLA and the SSC that are independent of defeat. There may be other sex differences not captured by the current study, such as in hormonally responsive brain regions like the central amygdala or the bed nucleus of the stria terminalis (BNST) (Ciccarelli et al. 2021). Future studies should examine potential sex differences more broadly. The present data strongly suggest that studies examining PNNs should include both sexes and be adequately powered to detect sex differences. In addition, in the present study, we chose to evaluate PNN expression on Diestrus Day 1 of the estrous cycle to try to minimize potential hormonal variation among the females. It is certainly possible that there is variation in PNNs over the estrous cycle and that there could be varying sensitivity to stress among females at different stages of the cycle that could influence the PNN response. These factors should certainly be considered in future studies seeking to fully understand sex differences in PNN expression.

In conclusion, we characterized PNN expression in Syrian hamster brains and suggested that hamsters may exhibit a unique distribution of PNNs compared to other commonly studied laboratory rodents. In addition, we have shown that there are marked sex differences in PNN expression in brain regions that are known to be involved in stress‐responding and perhaps in disordered responses to stress in patients with neuropsychiatric illnesses (Shin and Liberzon 2010). Furthermore, we have demonstrated that PNN expression in hippocampal area CA1 may be sensitive to social stressors in a sex‐dependent manner. Together, these data suggest that Syrian hamsters are a valuable model organism with which to examine the potential functional role of PNNs in behavioral responses to stress in males and females.

## Author Contributions

**Emma K. Shaughnessy**: conceptualization, investigation, writing–original draft, methodology, validation, visualization, writing–review and editing, formal analysis. **Benjamin W. Horne**: conceptualization, investigation, methodology, visualization. **Kim L. Huhman**: funding acquisition, investigation, conceptualization, writing–review and editing.

## Conflicts of Interest

The authors declare no conflicts of interest.

### Peer Review

The peer review history for this article is available at https://publons.com/publon/10.1002/brb3.70189.

## Data Availability

The data that support the findings of this study are available from the corresponding author upon reasonable request.
